# On Delirium Tremens

**Published:** 1846-12

**Authors:** 


*On Delirium Tremens.* By Dr. Corrigan, Dublin.—The following remarks by Dr. Corrigan on delirium tremens, present distinctions, which, although they have been described by other practical teachers, are perhaps not sufficiently considered by the profession at large. It is in a measure owing to the different circumstances and pathological conditions under which the disease may be developed, that such different methods of treatment in this disease have been recommended. The measures which in one case will be appropriate and successful, may be injudicious and hurtful in another case. It is the part of the physician to discriminate, and adjust his treatment to the particular circumstances and conditions of individual cases. We are indebted for the article to the British American Medical and Physical Journal.—*Ed. Buff. Jour.*

"A man comes under treatment, not after a few days' illness, but for a considerable time he has been subject in the morning to symptoms similar to those that follow the exhibition of large doses of opium, or of stimulants—symptoms, in fact, resembling collapse. His stomach is sick in the morning, the skin is clammy, and he is unable to collect his mind for any purpose until the accustomed stimulus is renewed. In this way he continues for an uncertain period of time, till at length vomiting sets in; he can no longer drink, and now the state of collapse, or nervous irritation, such as precedes an attack of acute disease, comes on, and obliges the patient to seek advice. He cannot sleep; images of various kinds float before the eyes; his stomach is sick; pulse quick and weak; skin cold and clammy—a set of symptoms constituting, as I have said, a state of collapse consequent on the cessation of long-continued stimulants. Your patient is altogether in a condition in which death may occur at any moment, so that the prognosis here is extremely uncertain.

In these cases it is necessary to give stimulants and opium; the opium, you are to remember, is given to allay irritation, and the stimulants in order to bear up the system. Cold douching and a variety of other remedies may be used; but upon them it is not necessary to dwell, as they are sufficiently noticed in every book you meet with. There is one particular symptom observed, usually I think about the second or third day, and one which is never absent; I allude to the tremulous motion of the fibres of the tongue; not of the whole body of the organ, but of a sort of independent motion of individual fibres here and there. The same thing is seen in the orbicularis muscle of the mouth. These signs are sufficient to lead us to a knowledge of the previous history, though the patient himself should deny the circumstances. It often happens that the patient has received a wound in some way; for instance thrusting his hand through a pane of glass. Now, if while you are giving the opium, you watch the appearance of such a wound, and find that, instead of secreting the natural purulent discharges, the edges are everted and red, with the surface dry, you may be assured your patient will not recover; for these indications, like those of the tongue, only being more certain, are evidences that the nutritive function is arrested, and life cannot long continue when that function is impaired to a great extent. Such, then, is a sketch of the more frequent form of the disease.

The next variety gets a similar name—delirium tremens—but we should carefully mark the distinctions between this and the first described variety, or a fatal mistake may be made. And here I should observe, that it is the fact of thus erroneously describing under one name varieties in this disease, which has given rise to such a contrariety of opinions respecting the mode of treatment, &c., proper to be adopted in it. Dr. Lendrick, a man of great observation, first showed that the ordinary treatment, as opium and stimulants, would not do here, and that bleeding should be had recourse to. I believe, then, that two very different conditions of disease have been confounded under the same name; so you are not always to suppose, when you have got hold of a name, that you have, by any means, got hold of the disease.

The case I am now about to describe may be called sthenic delirium tremens. A man has been drinking for two, three, or four days, and is in a condition very different from the person who has been a long time accustomed to stimulants; there is in this man a state of irritation of the brain and nervous system only in a very trifling degree removed from actual phrenitis, and were you to give opium in such a case, it would act, not as a sedative, but as a continued stimulant, and you would thus be keeping up the diseased condition into which the patient has plunged himself. Remember, then, that this is a mild case, there being a certain amount of irritation, but a strong approach to positive inflammation. Gastritis is a common accompaniment of this form of delirium tremens, at least a state, like that of the brain, of approaching inflammation of the stomach, marked, as I have said, by some symptoms of nervous irritation, but nothing like the collapse of the former case. During the period of a general election, cases of the sthenic kind are frequently met with; for instance, a man of previous abstemious habits spends two or three successive nights drinking ardent spirits, and presents himself with the symptoms I have sketched for you.

In this, the sthenic form of the disease, then, do not give opium; apply leeches to the epigastrium, and to the head, as also cold lotions; these, with rest and small doses of mercury, are your chief remedies. When you have to some extent allayed the irritation, you may then make a slight approach to the treatment adapted to the first case, but do not commence by stimulating. Recollect that a compound form the disease presents itself in this case. We now come to the third division of the disease, a form of it in which very little active treatment is necessary; and I may tell you that the skill of the medical man is often most seen in his abstaining entirely from any decided treatment; good practice consists in that as much as in any thing else. You must not entirely refrain however, from giving medicine, if it were merely for the purpose of keeping in view the advantage of the impression on your patient's mind that you are making some exertion for him: and this is a point deserving your attention, for were your patient even a medical man, it would be necessary to act on this principle.

A man presents himself, who has been at one time temperate, at another drinking perhaps for two or three days, and is now laboring under more or less irritation of the brain, manifested by slight attacks of delirium, and want of sleep, forming, in fact, a link between the condition already described as asthenic delirium tremens and the state of collapse. The subject of a case like this gets repeated attacks of a trifling kind; he may be as I have just said, at one time temperate, at another—perhaps in travelling and stopping at different hotels—drinking three, four, or a half-a-dozen days, and at length falls into a state constituting our third division; he is capable of exertion, understands what you say to him, and will speak collectively, but when left to himself, fancies strange sights hovering about him. He is neither, as I have said in the state of the collapse of the asthenic form, nor does he betray the symptoms of cerebral derangement observed in the sthenic variety of the disease, but there is danger of the affection assuming the perfect form from the too frequent recurrence of these slight attacks of mental aberration. The patient takes little or no nourishment. This variety of the disease, then, forms as it were the center of the balance–but let nature have the management of it; leeching will not be borne, from the previous habits of the patient, and if opium be given, so as to be followed by dryness of the tongue, great mischief is done; so do as little as possible. Give the patient as much cold air as you can, at the same time keeping his room rather dark. You may either give him ice, or a very cool saline mixture, or cold chicken tea. If restlessness should still persists, you may give small doses of opium, but much of the after-treatment had better be left to nature. The three divisions which I have sketched, you will find worth recollecting; they are such, as in an ordinary exercise of observation, in practice must force themselves upon you.—*London Med. Times.*

